# Patient understanding of the revised USPSTF screening mammogram guidelines: need for development of patient decision aids

**DOI:** 10.1186/1472-6874-12-36

**Published:** 2012-10-10

**Authors:** Summer V Allen, Lise Solberg Nes, Mary L Marnach, Kristen Polga, Sarah M Jenkins, Julia A Files, Ivana T Croghan, Karthik Ghosh, Sandhya Pruthi

**Affiliations:** 1 Department of Family Medicine, Mayo Clinic, 200 First Street SW, Rochester, MN 55905, USA; 2 Department of Medicine, University of Minnesota, D612 MMC 741, 420 Delaware Street SE, Minneapolis, MN 55455, USA; 3 Department of Obstetrics & Gynecology, Mayo Clinic, 200 First Street SW, Rochester, MN 55905, USA; 4 Division of Internal Medicine, Marshfield Clinic, 1000 North Oak Avenue, Marshfield, WI 54449, USA; 5 Department of Health Sciences Research, Mayo Clinic, 200 First Street SW, Rochester, MN 55905, USA; 6 Division of Women’s Health Internal Medicine, Mayo Clinic, 13737 North 92nd Street, Scottsdale, AZ 85260, USA; 7 Primary Care Internal Medicine, Nicotine Research Program, Mayo Clinic, 200 First Street SW, Rochester, MN 55905, USA; 8 Division of General Internal Medicine, Mayo Clinic, 200 First Street SW, Rochester, MN 55905, USA

**Keywords:** Anxiety, Confusion, Guidelines, Health status, Screening mammogram, USPSTF

## Abstract

**Background:**

The purpose of the study was to examine patients’ understanding of the revised screening mammogram guidelines released by the United States Preventive Services Task Force (USPSTF) in 2009 addressing age at initiation and frequency of screening mammography.

**Methods:**

Patients from the Departments of Family Medicine, Internal Medicine, and Obstetrics and Gynecology (n = 150) at a tertiary care medical center in the United States completed a survey regarding their understanding of the revised USPSTF guidelines following their release, within four to six months of their scheduled mammogram (March 2010 to May 2010).

**Results:**

Of the patients surveyed, 97/147 (67%) indicated increased confusion regarding the age and frequency of screening mammography, 61/148 (41%) reported increased anxiety about mammograms, and 58/146 (40%) reported anxiety about their own health status following the release of the revised screening guidelines. Most of the patients surveyed, 111/148 (75%), did not expect to change their timing or frequency of screening mammograms in the future.

**Conclusion:**

Results from this survey suggested increased confusion and possibly an increase in patients’ anxiety related to screening mammography and their own health status following the release of the revised USPSTF screening mammogram guidelines to the public and subsequent media portrayal of the revised guidelines. Although the study did not specifically address causality for these findings, the results highlight the need for improvements in the communication of guidelines to patients and the public. Development of shared decision-making tools and outcomes should be considered to address the communication challenge.

## Background

Breast cancer is the second most common cause of cancer mortality among women in the United States [1,2]. Screening mammography has been an important tool in early detection of breast cancer, resulting in a decrease in breast cancer mortality [3,4]. In November 2009, the United States Preventive Services Task Force (USPSTF), an independent panel of experts in prevention and primary care, released a revision of the screening mammogram guidelines pertaining to the age at which to initiate mammography and the frequency of screening mammography. The guidelines had last been updated in 2002.

The USPSTF suggested that screening mammography leads to the greatest absolute reduction in breast cancer mortality in women aged 50 to 74 years. The following were listed as potential risks from screening mammography: psychological harm, additional medical visits, imaging and biopsies in women without cancer, inconvenience from false-positive screening results, and the potential harm of unnecessary treatment and radiation exposure [5-7]. The USPSTF revised guidelines were published in the Annals of Internal Medicine in November 2009 and recommended against routine screening mammography for women aged 40–49 years, suggesting that routine screening mammography begin at age 50 and continue biennially [7]. The recommendation for biennial screening after age 50 was based on modeling data that indicated biennial rather than annual screening confers an improved benefit-risk ratio [8]. The announcement of these revised recommendations created significant public controversy [9,10], and the USPSTF, in their testimony to the Subcommittee on Health of the House Energy and Commerce Committee soon after the announcement, acknowledged that communication about the recommendations could have been better [11]. Subsequently, the USPSTF unanimously voted to clarify the language in their previous statement. On December 4, 2009, the USPSTF clarified their communication and the focus was placed on the second sentence which stated, “The decision to start regular, biennial mammography screening before age 50 years should be an individual one and should take patient context into account, including the patient’s values regarding specific benefits and harms” [5].

The revised USPSTF recommendations received the support of organizations such as the American College of Physicians [12] and the American Academy of Family Physicians [13], generally based on the notion that the revised guidelines may help decrease perceived excessive screening leading to over-diagnosis, unnecessary treatment, and potential psychological stress. Other organizations, such as the American College of Obstetrics and Gynecology, American Cancer Society, National Comprehensive Cancer Network, American College of Radiology, and the Susan G. Komen for the Cure, continue to recommend initiating annual screening mammography at age 40 [7,14-16]. The USPSTF also recommended against routine screening to avoid characterizing all women the same way and emphasized an individualized approach for women. The importance of informed decision making regarding screening mammography has been a point of agreement between supporters and critics of the revised guidelines [17,18]. The lack of available tools to aid in decision making provides a challenge to patients and practitioners seeking to make an informed decision in this context [7,10,16,19].

The revised guidelines were also subjected to significant media scrutiny and criticism in the weeks immediately following publication of the guidelines in 2009 [9,10,20]. A recent study examining media content and patient knowledge about the revised recommendations demonstrated that the majority of newspaper articles and social media posts published within the first two months of the release of the revised guidelines were unsupportive, stating that women were confused by the recommendations [20].

The current study sought to investigate the impact of the release of the revised 2009 USPSTF screening mammogram guidelines on patient understanding of the recommended age and frequency of screening mammography. The study also aimed to examine the patients’ plans related to decision making about future mammography for breast cancer screening.

## Methods

### Study population

The study was conducted in a subset of female patients receiving continuity care within the Departments of Family Medicine, Internal Medicine, and Obstetrics & Gynecology at a tertiary care medical center between March 25, 2010 and May 31, 2010. Female patients (n = 304) between the ages of 40 and 75, who were due for health maintenance or gynecologic examination during the two-month period, were selected at random through computerized selection. Selected patients were mailed a letter and survey inviting their participation in the study. Patients who did not respond to the first mailing received a second mailing. Those who did not return a completed survey after the second mailing were classified as nonresponders. The Mayo Clinic Institutional Review Board approved the study prior to recruitment.

### Measures

Through careful consideration by the authors and input from the Mayo Clinic Institutional Review Board and survey specialists, a survey was created to assess the impact of the release of the revised 2009 USPSTF screening mammogram guidelines. The survey focused on patient awareness, interpretation, feelings (measured on a Likert scale), and future plans regarding screening mammography. Topics of interest included (see Additional file 1):

Demographics, screening mammography status, and family history

Understanding of the guidelines and how they received information about the revised screening mammogram guidelines

Understanding of screening recommendations

Impact of revised screening mammogram guidelines on feelings related to screening and personal health status (health anxiety)

State anxiety

Personal and family history related to breast cancer, and awareness of institutional position on the revised guidelines

### Statistical analyses

Our primary goal was to describe the distribution of the responses of a patient population to the survey questions. Categorical survey responses were summarized with percentages and 95% confidence intervals score, and age was summarized with use of mean and range.

A secondary goal was to examine potential differences among the women invited to participate in the study. Responses were compared between demographic characteristics using the Fisher exact test. Respondents were compared to nonrespondents with respect to age (2-sample *t*-test) and county residency (chi-square test) to see whether there may have been response bias with respect to these characteristics. Comparisons yielding p-values less than 0.05 were considered statistically significant. All analyses were performed using SAS version 9 (SAS Institute Inc., Cary, NC) [21].

## Results

### Patient characteristics

Of the 304 patients who were sent a survey, 150 patients (approximately 50%) responded. Responders and nonresponders did not differ significantly between age and residence. Among the 150 responders, the majority were white (96%), mean age 56 years (range 40–75), and were in the age group of 50 to 75 years old (n = 91). Most of the patients (61%) reported living in counties adjacent to the medical center and only 13% reported living outside of the state. All (100%) surveyed participants had completed high school; 25% had completed college and 12% had a graduate degree. Most patients (97%) reported currently receiving annual screening mammograms and 49% reported having had more than 10 previous mammograms.

### Awareness of the revised 2009 USPSTF guidelines

Of the respondents, 62% were somewhat aware and 25% were very aware of the revised USPSTF guidelines; only 13% reported being unaware of the revised guidelines. The majority of patients (92%) reported that they had learned about the guidelines through the news media and the remaining participants learned through internet (16%), health care provider (12%), family and friends (11%), books and magazine (7%), and other sources (3%). Patients (54%) also indicated they were unaware of their medical institution’s position on the new USPSTF guidelines.

### Understanding of the revised guidelines

In general, a majority of the patients understood and were in agreement with the general breast cancer screening recommendations in place before the release of the revised USPSTF guidelines (Table 1). Understanding was less apparent among participants after the release of the revised guidelines (Table 1). Most participants reported not being confident (46%) or somewhat confident (41%) about their understanding of the revised guidelines.

**Table 1:** Patient understanding of screening recommendations before and after the release of the revised guidelines

	n* (%, 95% Confidence Interval)	
**Survey question**	**Before release**	**After release**
What was your understanding regarding the age at which most women should begin screening mammograms?		
Under 40 years old	23 (15.3, 10.4-22.0)	7 (4.7, 2.3-9.4)
40 to 49 years old	118 (78.7, 71.4-84.5)	22 (14.9, 10.0-21.5)
50 to 60 years old	5 (3.3, 1.4-7.6)	85 (57.4, 49.4-65.1)
Over 60 years old	1 (0.7, 0.1-3.7)	5 (3.4, 1.5-7.7)
Not sure	3 (2.0, 0.7-5.7)	29 (19.6, 14.0-26.7)
What was your understanding of how frequently it is recommended that women have screening mammograms between 40 and 50 years of age?		
No screening	0	43 (28.9, 22.2-36.6)
Yearly	119 (79.3, 72.2-85.0)	11 (7.4, 4.2-12.7)
Every other year	17 (11.3, 7.2-17.4)	26 (17.4, 12.2-24.3)
Every three years or more	8 (5.3, 2.7-10.2)	19 (12.8, 8.3-19.1)
Not sure	6 (4.0, 1.8-8.5)	50 (33.6, 26.5-41.5)
What was your understanding of how frequently it is recommended that women have screening mammograms 51 years and older?		
No screening	1 (0.7, 0.1-3.7)	2 (1.3, 0.4-4.8)
Yearly	126 (84.0, 77.3-89.0)	26 (17.4, 12.2-24.3)
Every other year	8 (5.3, 2.7-10.2)	40 (26.8, 20.4-34.5)
Every three years or more	4 (2.7, 1.0-6.7)	21 (14.1, 9.4-20.6)
Not sure	11 (7.3, 4.1-12.7)	60 (40.3, 32.7-48.3)

*Totals not adding to 150 indicate missing data (not included in percentage calculation).

### Emotions related to revised guidelines

A majority of patients (66.7%) reported being more confused following the release of the revised guidelines (Table 2). Additionally, while most patients reported no or minimal state anxiety (i.e., current general anxiety at the time of survey completion), a number of the patients reported feeling more anxious as a result of the revised screening guidelines. Greater than 50% of the patients also reported more anxiety about their health status following the revised guidelines (Table 2).

**Table 2:** Impact of new screening guidelines on patients: confusion and anxiety

**Survey question**	n* (%, 95% Confidence Interval)	
The new USPSTF screening guidelines/recommendations have made me feel:		
Less confused about screening mammograms	4 (2.7, 1.1-6.8)	
No change about screening mammograms	45 (30.6, 23.7-38.5)	
More confused about screening mammograms	71 (48.3, 40.4-56.3)	
Much more confused about screening mammograms	27 (18.4, 12.9-25.4)	
Please note how anxious you feel at the moment (“state anxiety”).		
Not at all anxious	84 (56.8, 48.7-64.5)	
A little anxious	35 (23.6, 17.5-31.1)	
Moderately anxious	15 (10.1, 6.2-16.0)	
Very anxious	12 (8.1, 4.7-13.6)	
Extremely anxious	2 (1.4, 0.4-4.8)	
The new USPSTF screening guidelines/recommendations have made me feel regarding screening mammograms and health status:	Screening mammograms	Health status
Much less anxious	2 (1.4, 0.4-4.8)	2 (1.4, 0.4-4.9)
Less anxious	4 (2.7, 1.1-6.7)	2 (1.4, 0.4-4.9)
No change	81 (54.7, 46.7-62.5)	84 (57.5, 49.4-65.3)
More anxious	50 (33.8, 26.7-41.7)	46 (31.5, 24.5-39.4)
Much more anxious	11 (7.4, 4.2-12.8)	12 (8.2, 4.8-13.8)

Abbreviation: USPSTF, United States Preventive Services Task Force.

*Totals not adding to 150 indicate missing data (not included in percentage calculation).

### Family history of breast cancer

Among the 150 responding patients, 57 (38%) reported a family history of breast cancer (of the 38%: 40% reported mother, 19% reported sister, 2% reported daughter, and 63% reported other); 62% of the patients had no such family history. There was no significant difference in the lifetime number of mammograms between women with and without a family history of breast cancer. More patients with a family history of breast cancer than those without (35% vs. 18%) reported being very aware of the revised screening guidelines (p < .05). There were, however, no significant differences in these groups with regards to state anxiety about screening mammograms or health status following the release of the revised USPSTF guidelines.

### Decision regarding future plans for screening mammograms

Of the responding patients, 75% did not expect to change the age at initiation or frequency of screening mammography in the future, whereas 25% either expected to change or did not know if they would alter their screening behavior (Table 3). Between these groups, there was a significant difference in the understanding of how frequently it is now recommended that women have screening mammograms between 40 and 50 years of age (p = .02). Uncertainty of the revised guidelines was greater among women planning to change their screening practices versus those who were not planning to change (54% vs. 25%). There was also a significant difference between these groups on reported anxiety related to screening mammograms (p = .03), with patients expecting to change their practice (or uncertain of whether to do so) reporting significantly more anxiety (54% vs. 27%). No significant difference was found between these groups with respect to anxiety regarding their health status.

**Table 3:** Patient intent to change practice

**Survey question**	n* (%, 95% Confidence Interval)
As a result of the new USPSTF screening guidelines/recommendations, are you expecting to change the time and/or frequency of when you receive your screening mammograms in the future?	
No	111 (75.0, 67.5-81.3)
Yes	7 (4.7, 2.3-9.4)
Not sure	30 (20.3, 14.6-27.5)

Abbreviation: USPSTF, United States Preventive Services Task Force.

*2 missing responses among patients (not included in calculations of percentages).

## Discussion

Preventive care is an important component of health care. New or revisions to preventive care guidelines, especially when controversial, present immense challenges to patients as well as providers as they influence the education of patients and the shared decision-making process. In this study, we found that most of the patients learned of the revised guidelines through the media. A majority of the women in the survey study reported they were confused and anxious after the release of the revised guidelines and unsure about how the changes would impact their current screening schedule.

Despite reporting awareness of the revised guidelines, a large number of patients described low confidence in their understanding of the revised guidelines. In addition, more patients were in agreement with existing recommendations about screening mammography before the release of the revised guidelines than after the release. A large number of responders also described increased confusion following the release of the revised guidelines likely secondary to the change in recommendations, as well as news presentations and publicized disagreements among major health organizations [20]. As noted in the commentary by Woolf in JAMA, unless guideline-related challenges are resolved, there will be a lack of understanding and confusion about how and when to pursue clinical preventive services [9].

In addition to reported confusion regarding the age at which to initiate mammography as well as the frequency of screening, many of the patients also described feeling more anxious about mammography screening and about their own health status as a result of the change in screening guidelines. This is striking, particularly because a majority of the participating patients (80%) reported very little or no anxiety (i.e., state anxiety) at the time of the survey completion. These findings emphasize the need for improved communication of the role of screening mammography and its impact on a woman’s health.

A majority of the patients in the current study did not expect to change their age of initiation of mammography and frequency of screening despite the revised guidelines. Patients expecting to change their screening practice did, however, report significantly more anxiety about screening mammograms than their counterparts. It is generally expected that a family history of breast cancer will impact the willingness of patients to engage in frequent screening. In the current study, patients with a family history of breast cancer reported higher awareness of the new screening guidelines. There were, however, no significant differences between these groups with regard to frequency or number of previous mammograms, nor any significant differences with regard to current state anxiety about screening mammograms, or anxiety about health status following the release of the revised USPSTF guidelines. It is possible that patients with a family history of breast cancer have a greater knowledge and understanding of the role of screening mammograms although they too could benefit from shared decision-making tools.

This study showed that women have experienced confusion, anxiety, and lack of confidence in the current screening guidelines since the release of the revised USPSTF screening mammogram. Although causality for these findings can be multifactorial, it is not clearly known whether it was the revised USPSTF guidelines or the media portrayal of the guidelines that contributed to the increasing confusion. Other factors contributing to patient confusion may have been the variable opinions of clinicians when communicating the issue of the revised guidelines, as well as the short time interval between the release of the new recommendations and this study.

The confusion and anxiety reported by the patients in the current study highlight a pronounced need to improve the communication and presentation of information to patients. The revised guidelines emphasize the importance of individual risk assessment and desirability of shared decision making with patients, particularly with regards to mammogram screening for women 40–49 years of age. Organizations that create or change guidelines may consider simultaneously creating decision-making tools, which can be used to communicate appropriate and relevant information to improve patient understanding of guidelines, that address the issues at hand and individual needs of the patient. Decision aids used in patient education have been shown to be effective and to improve patient understanding of options and potential outcomes, and there is research available providing suggestions for guidelines and quality criteria frameworks for the development and use of patient decision aids [22]. For example, in Australia and Canada, mammography screening decision aids have already been created, and in Australia the new shared decision-making tool is currently undergoing a clinical trial [23,24].

Necessary for shared decision making is understanding personal risk, and unfortunately, conveying the concept of risk to a patient is often challenging [25]. Decision-making tools could help to reduce anxiety by providing a concise review of the risks, benefits, and limitations of screening mammograms and also help clarify important options [26].

The current study aimed to capture the perceived impact of the release of the revised USPSTF screening mammography guidelines for a subgroup of patients shortly after the information about the new guidelines were provided to the public. Because existing validated surveys reflecting issues raised by the revised USPSTF screening mammogram guidelines were not available, a new survey was designed to address the specific aims of this study. The survey post-test design makes it difficult to determine causality of the results. However, the survey questions specifically focused on impact (e.g., “The new USPSTF screening guidelines have made me feel…”) and clearly relates to this issue. Participants in this study were primarily white and associated with a single medical center that supported annual screening mammograms beginning age 40. These factors could potentially influence generalizability of our findings. In addition, local and national media reports on the topic may have introduced bias. However, the patients in this study did not receive a direct mailing from their health care providers related to breast cancer screening, and even though a majority of the participants reported learning about the new guidelines through the media, most responders denied being aware of the institution’s position on the revised guidelines. The results from this study may possibly represent the general public’s understanding and impact of the release and presentation of the revised guidelines.

## Conclusions

This study demonstrated that since the release of the revised USPSTF screening mammogram guidelines, women have experienced confusion, anxiety, and lack of confidence in the current screening guidelines. It is not clearly known whether it was the revised USPSTF guidelines or the media portrayal of the guidelines that contributed to the increased confusion. Even when based on the same evidence, cancer screening guidelines can differ among different organizations and can create confusion among the public, health care providers, and policy makers. Findings from this study emphasize the importance of a systematic approach to the development of screening guidelines and communication to the public in particular, as the choices regarding cancer screening involve more complex psychological, scientific, and financial factors [27]. Prudence would dictate that in order to make informed decisions, patients should receive information describing risks, harms, and benefits of screening. The development of shared decision-making tools, as well as appropriate endpoints or outcomes of such tools, should be considered to address communication challenges that may arise from the release of new guidelines. These tools should be individualized to aid women when counseling and educating them about the utilization of screening mammograms.

## Competing interests

The authors declare that they have no competing interests.

## Authors’ contributions

SP, SVA, LSN, and SMJ participated in the conception, design, acquisition of data, analysis, interpretation of the data, and drafting the manuscript. JAF, KG, ITC, KP, and MLM were involved with revising the manuscript critically for important intellectual content. All authors have read and approved the final manuscript.

## Pre-publication history

The pre-publication history for this paper can be accessed here:

http://www.biomedcentral.com/1472-6874/12/36/prepub

## Supplementary Material

Additional file 1 Mammography Survey. Click here for file
