# Supplementary material for: Patient understanding of the revised USPSTF screening mammogram guidelines: need for development of patient decision aids
Source: BMC Womens Health. 2012 Oct 10;12:36. doi: 10.1186/1472-6874-12-36 (PMC3517367; doi:10.1186/1472-6874-12-36)
Supplement: Additional file 1 — Mammography Survey. [file 1472-6874-12-36-S1.doc]

**Additional file 1**

Recent guidelines/recommendations regarding mammography have been made by the United States Preventive Services Task Force (USPSTF). Based on this information, please answer the following questions to the best of your knowledge:

1. What is your awareness of the new screening mammogram guidelines/recommendations that were released in November 2009 by the USPSTF? *(scale of 1-3; if aware, how did you learn about this information?)*

For each of the following three questions, please indicate what your understanding was before and after the release of the USPSTF guidelines/recommendations.

2. Before/Since the release of USPSTF guidelines/recommendations, what was your understanding regarding the age at which most women should begin screening mammograms? *(list of age choices)*

3. Before/Since the release of USPSTF guidelines/recommendations, what was your understanding of how frequently it is recommended that women have screening mammograms between 40 and 50 years of age? *(list of screening frequencies)*

4. Before/Since the release of USPSTF guidelines/recommendations, what was your understanding of how frequently it is recommended that women have screening mammograms 51 years and older?
*(list of screening frequencies)*

5. The new USPSTF screening guidelines/recommendations have made me feel: *(scale of 1-5)*

6. Please note how anxious you feel at the moment. *(scale of 1-5)*

7. The new USPSTF screening guidelines/recommendations have made me feel: *(scale of 1-5 regarding anxiety about mammograms)*

8. The new USPSTF screening guidelines/recommendations have made me feel: *(scale of 1-5 regarding personal health status)*

9. How confident are you that you understand the new USPSTF screening guidelines/recommendations:
*(scale of 1-5)*

10. As a result of the new USPSTF screening guidelines/recommendations, are you expecting to change the time and/or frequency of when you receive your screening mammograms in the future? *(yes/no/unknown)*

11. What is your age?

12. Do you live in Rochester? *(yes/no; if no, where?)*

13. What is your racial ethnic background?

14. What is the highest level of education you have completed?

15. Have you been getting annual screening mammograms? *(yes/no; if yes, how many/how often?)*

16. Does anyone in your family have a history of breast cancer? *(yes/no; if yes, which family members?)*

17. What is your awareness of Mayo Clinic’s position on the new USPSTF guidelines? *(scale of 1-3; if aware, what is your understanding of Mayo Clinic’s position [supports/doesn’t support] on the new USPSTF guidelines?)*
